# Current Advances in Molecular Phylogenetics

**DOI:** 10.1155/2014/596746

**Published:** 2014-04-07

**Authors:** Vassily Lyubetsky, William H. Piel, Dietmar Quandt

**Affiliations:** ^1^Institute for Information Transmission Problems (Kharkevich Institute), Russian Academy of Sciences, Moscow 127994, Russia; ^2^Yale-NUS College & National University of Singapore, Singapore; ^3^Rheinische Friedrich-Wilhelms-Universität, Bonn, Germany

Since its inception some 50 years ago, phylogenetics has permeated nearly every branch of biology. Initially developed to classify objects based on a set of cladistic rules, it has now become the central paradigm of evolutionary biology and a key framework for making sense of a wide range of disciplines [1], such as genomics [2], community ecology [3], epidemiology [4], conservation biology [5], and population dynamics [6], to name just a few. It is a testament to the power of phylogenetic methods that its application has expanded far beyond its original inception, now including the study of human culture, such as language and cultural memes [7].

Phylogenetic principles are used to reconstruct complex ancestral traits of morphological characters, genome structures and their properties, and evolutionary events (like gene duplications, losses, transfers, or chromosomal rearrangements). Phylogeny is also essential to infer gene and protein families, uncover complex population histories in epidemiological and other studies, and understand viral and cell genealogies in medicine and developmental biology. New concepts are developing that tackle various aspects of coevolution, including approaches to defining and algorithmically constructing complex evolutionary scenarios for genetic systems, their regulations, epigenetic and intrinsic factors, noncoding genome elements, sequence primary and secondary structures, the speciation process, and so forth.

The growth of phylogenetics is not just in breadth of disciplines, but also in the sheer volume of published phylogenetic results. Some twenty years ago, near-exponential growth in phylogenetic publications had already been noticed [8, 9], a growth that was probably attributable to the advent of powerful computers, PCR, and Sanger sequencing. An update on the assessment of phylogenetic growth (Figure 1) shows that not only is the growth in phylogenetic papers exponential, but more importantly the growth in the percentage of papers that report phylogenetic results is also exponential, indicating its increasing share in scientific research. Journals and databases have worked hard to keep pace with this growth, with the development of data repositories to archive and share data (e.g., TreeBASE, http://treebase.org/ and Dryad, http://datadryad.org/) that would otherwise be inefficient to distribute as supplementary addenda.

In the last ten years, the rate of growth of phylogenetic publications has waned somewhat (Figure 1), but with the recent advent of next-generation sequencing (NGS) we anticipate a new flood of phylogenetic results that is commensurate with this explosion of NGS data In addition to the phylogenetic results themselves, we also anticipate the need for new methodological papers to improve efficiencies in sequence assembly, multiple alignment, genome annotation, and pipelining of massive analyses.

Computational power is at risk of being outstripped because the volume of NGS data more than doubles each year, outpacing Moore's Law [10]. The limits of computational power portend the need for novel analytical approaches [11], among them “exact models” that avoid heuristics by finding mathematically provable global optima for a function, yet requiring low polynomial complexity, developing effective supertree and divide-and-conquer methods. Other perspective directions include modeling of coevolution as a system of stochastic processes, low-polynomial methods of simultaneous phylogeny and alignment construction, and applying mathematically proved methods to simulate test datasets for benchmarking phylogenetic algorithms. These anticipated advances also need new publishing avenues for dissemination to the scientific community.

This special open-access issue, which we hope to be an annual occurrence with *Biomed Research International*, seeks to meet the anticipated demand for disseminating phylogenetic results and phylogenetic methods. The special issue covers a variety of topics in modern phylogenetics and its applications, from phylogenetic systematics to new methodological developments and reviews. Many authors of the special issue also contributed to the Moscow Conference on Molecular Phylogenetics (http://www.en.molphy.ru/), which is organized biannually by Moscow State University and the Institute for Information Transmission Problems of the Russian Academy of Sciences. The call for papers for the next issue “Molecular Phylogenetics 2014” is now open, and we believe that this series will serve as a platform to exchange ideas and publish research in this actively expanding interdisciplinary field.

## Figures and Tables

**Figure 1:**
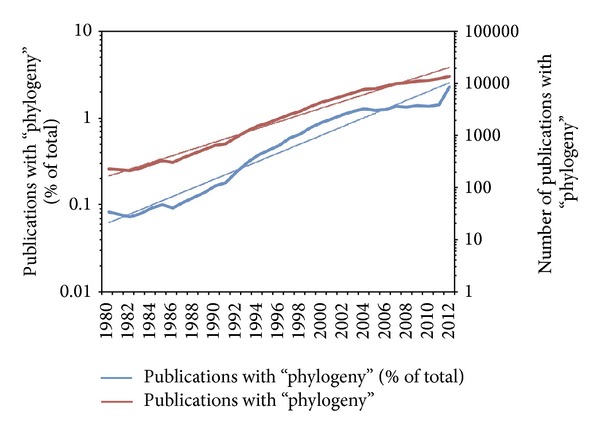
Growth of phylogenetic publications 1980–2012. Both the number of publications that involve “phylogeny” or “phylogenetic” terms and the proportion of publications appear to grow in a way that approximates exponential growth. Data were compiled from PubMed (http://www.ncbi.nlm.nih.gov/pubmed).
